# Study of the Antimalarial Activity of the Leaf Extracts and Fractions of *Persea americana* and *Dacryodes edulis* and Their HPLC Analysis

**DOI:** 10.1155/2021/5218294

**Published:** 2021-07-17

**Authors:** Philip F. Uzor, Chukwuebuka K. Onyishi, Adaeze P. Omaliko, Somtochukwu A. Nworgu, Onyemaechi H. Ugwu, Ngozi J. Nwodo

**Affiliations:** Department of Pharmaceutical and Medicinal Chemistry, University of Nigeria, Nsukka 410001, Enugu, Nigeria

## Abstract

In the present study, the antimalarial activity of the extracts and fractions of the leaves of *Persea americana* and *Dacryodes edulis* as well as their phytochemical compositions were examined. Each of the extracts of the plants was successively fractionated to obtain hexane, ethyl acetate, methanol, and water fractions. The extracts and fractions were tested against *Plasmodium berghei* in both curative and suppressive antimalarial mouse models. Their major phytochemical composition was studied by the standard chemical tests and HPLC analysis. The extracts and fractions of *P. americana* and *D. edulis* demonstrated significant (*p* < 0.05) maximal plasmodial inhibition as 52.16 ± 2.77% and 57.10 ± 1.98%, respectively, and chemosuppression of parasitemia as 64.01 ± 0.08% and 71.99 ± 0.06%, respectively. The major secondary metabolites identified in the plants include alkaloids, flavonoids, and saponins. It was concluded that *P. americana* and *D. edulis* possess promising antimalarial activity and they are potential sources of new lead compounds against malaria.

## 1. Introduction

Malaria is a major parasitic disease that is endemic in the tropics particularly in Africa [1, 2]. The disease has a great impact on the economy of the affected communities as there is associated loss of man-hours and consequent decline in the agricultural productivity of the people [3, 4]. In the year 2020, an estimated number of 229 million new cases of malaria and 409,000 deaths were reported by the WHO globally [5]. Nigeria is one of the countries in Africa where the disease causes a major healthcare problem [6, 7].

Not only are the conventional drugs often unaffordable or inaccessible but also the rate at which *Plasmodium falciparum,* the causative organism, has developed resistance to the conventional drugs warrants the quest for novel and more effective agents [8] particularly from natural resources. Medicinal plants have long been used for malaria treatment in many traditional settings [9, 10]. Two common ingredients in Nigeria ethnomedicine used for malaria treatment are the leaves of *Persea americana* and *Dacryodes edulis* which are used either singly or in combination.

*Persea americana* Mill. (Family: Lauraceae) is a plant that grows throughout the tropics and bears an edible fruit known as avocado. The seeds are used traditionally for the treatment of skin rashes, diarrhea, high blood pressure, dysentery, asthma, and rheumatism [11]. The plant is used in Nigerian [12, 13] and Guinean [14] ethnomedicine for the treatment of malaria. The antiprotozoal, antidiabetic, and anticonvulsant activities of the seed have been demonstrated [15, 16]. However, despite the reported use of the plant in ethnomedicines for malaria treatment, detailed investigations into the antimalarial potential of the plant have not been done [13].

*Dacryodes edulis* (G. Don) Lam (Burseraceae) is an evergreen tree that is traditionally used in several parts of Africa to treat various diseases including tonsillitis, sickle cell, skin diseases, and malaria [17, 18]. The analgesic, antiallergic, and antimicrobial activities of *D. edulis* have been investigated [17]. Moreover, the leaves of the plant have shown a significant *in vitro* antiplasmodial activity [19] while the stem bark extract and five isolated compounds were shown to have strong antiplasmodial activity *in vitro* [17].

Given the above, this study therefore aimed at assessing the antimalarial activity of *P. americana* and *D. edulis* extracts and fractions in mice as well as examining the major class of phytoconstituents of the plants.

## 2. Materials and Methods

### 2.1. Plant Materials

*P. americana* and *D. edulis* fresh leaves were collected within the University of Nigeria, Nsukka, and authenticated by Mr. Felix Nwafor of the Department of Pharmacognosy and Environmental Medicines, University of Nigeria, Nsukka, with the identification vouchers of PCG/UNN/0079 and PCG/UNN/0078, respectively. The plant materials were washed with distilled water, air-dried, powdered, and stored in the refrigerator prior to use.

### 2.2. Plant Extraction and Fractionation

Plant extraction and fractionation were done as previously reported with some modifications [20]. A certain quantity (500 g) of each of the powdered plants was weighed and macerated in 1.5 L of 95% methanol in water (BDH, England) at room temperature (28 ± 2°C) for 72 h with occasional agitation and daily washing. Each suspension was filtered and the filtrate concentrated using a rotary evaporator at 40°C to afford the crude extracts coded as PA (for *P. americana*) and DE (for *D. edulis*). Each of the crude extracts (DE and PA) was further subjected to fractionation by dissolving it successively with *n*-hexane, ethyl acetate, methanol, and water. This procedure afforded the respective fractions coded for *P. americana* and *D. edulis* respectively, as follows: PAH and DEH (for n-hexane), PAE and DEE (for ethyl acetate), PAM and DEM (for methanol), and PAW and DEW (for water) fractions.

### 2.3. Animals

Animals used for the study were albino mice of either sex and weight of 11–18 g. A total number of 126 mice were used for the experiments. The animals were obtained from the Faculty of Veterinary Medicine, University of Nigeria, Nsukka. The animals were acclimatized for 7 days before experimentation. They were given access to water and feed *ad libitum*. Animal handling was in accordance with the internationally accepted guidelines (EEC Directive of 1986; 86/609/EEC).

### 2.4. Acute Toxicity Studies

The testing for acute toxicity was done according to Lorke's method [21]. It was done in two stages: for the first stage, nine mice were selected and these segregated into three groups of three animals in each group. The first group received 10 mg/kg (p.o.) of the extract (PA or DE) while the second and third groups of the animals received 100 mg/kg and 1000 mg/kg of the extract (PA or DE), respectively. After dosing, the animals were kept under observation for 24 h to find out the mortality if any and also for the next 14 days. Based on the number of deaths in each group in the first stage, the doses (four selected doses ranging from 1 mg/kg to 5000 mg/kg) for the second stage were determined according to the Lorke's [21] schedule. The LD_50_ was calculated using the formula

(1) $$\begin{array}{c} {\text{LD}}_{50}=\sqrt{\text{Minimum toxic dose}\times \text{Maximum tolerated dose}}, \end{array}$$

where minimum toxic dose = the least dose that produced 100% death and maximum tolerated dose = the highest dose at which there was no death.

If no death was recorded in the second stage, the LD_50_ was taken as greater than the maximum dose administered.

### 2.5. Inoculation of Parasites

Blood was drawn from a donor mouse which has been infected with *P. berghei*. Blood was drawn by heart puncture and diluted to a final concentration of about 1 × 10^7^ infected red blood cells (RBC) per 0.2 ml suspension, following the procedure earlier reported [22].

### 2.6. Curative Antimalarial Test (Rane's Test)

The curative test was done as reported previously [23]. On day 0, standard inocula (0.2 ml) as prepared above were injected in mice intraperitoneally. About 72 h later (day 3), 45 mice were randomly segregated into nine (9) groups of five (5) mice and dosed (p.o.) once daily for five days (day 3 to day 7) according to the following groupings: groups 1–3 were parasitized and received, respectively, 100 mg/kg, 200 mg/kg, and 400 mg/kg of PA while groups 4–6 were parasitized and received, respectively, 100 mg/kg, 200 mg/kg, and 400 mg/kg of DE. Group 7 was parasitized and received the standard drug, ACT (artemisinin combination therapy), while group 8 (control group) was infected but untreated (received vehicle only). Parasitemia was monitored by preparing a thin blood film from the tail of each mouse on day 3 (before treatment) and day 8 by the use of Giemsa stain. The slides were examined under a microscope on ×100 magnification. Parasitemia was determined as follows [22]:

(2) $$\begin{array}{c} \text{\% Parasitemia }=\;\frac{\text{number of parasitized RBC}}{\text{total number of RBC }}\times 100\text{.} \end{array}$$

On day 8, mice were sacrificed and blood samples were collected for hematological (Hb and PCV) analysis.

### 2.7. The Four-Day Suppressive Test

Schizonticidal activity of the extracts and the fractions was tested by the 4-day suppressive test according to the method described elsewhere [24]. Fifty-five (55) infected mice were randomly divided into 11 groups (*n* = 5) as follows: group 1 animals were parasitized and received 400 mg/kg of PA while groups 2–5 were parasitized and received, respectively, 200 mg/kg each of PAH, PAE, PAM, and PAW. Similarly, for DE, group 6 animals were parasitized and received 400 mg/kg of DE while groups 7–9 were parasitized and received, respectively, 200 mg/kg of DEH, DEE, and DEM. Group 10 was parasitized and received ACT while group 11 (control group) was infected but untreated (received vehicle only). On day 0, standard inocula (0.2 ml) as prepared above were injected in mice intraperitoneally. A four-day treatment was started on day 0, 3 h after inoculation, and then continued once daily for the next three days (i.e., from day 0 to day 3). Thin blood film was prepared on day 4 and examined as described in Rane's test above. Percentage suppression of parasitemia was calculated as follows:

(3) $$\begin{array}{c} \text{\% Suppression }=\;\frac{\left(\text{mean parasitemia of control group}-\text{mean parasitemia of treated group}\right)}{\text{mean parasitemia of control group}}\times 100\text{.} \end{array}$$

Each mouse was also observed daily for the determination of the survival time and the mean survival time (MST) calculated arithmetically from the date of infection over a period of 30 days (day 0–day 29).

### 2.8. Determination of the Hematological Parameters

The PCV was determined using standard Micro-Hematocrit Reader as previously described [22]. Hb was measured using a cyanmethemoglobin technique [25]. Determination of the hematological parameters was done using the extracts (PA and DE) only.

### 2.9. Preliminary Phytochemical Analysis

Preliminary phytochemical tests for the presence of the major class of phytoconstituents were conducted following standard procedures [20].

### 2.10. HPLC Analysis

HPLC (Dionex P580®) framework with a photodiode detector (UVD340S) was employed for the HPLC investigation. MeOH and 0.02% H_3_PO_4_ in H_2_O were utilized as mobile phase. Peak identification was ascertained by comparing their ultra-violet (UV) spectra with those on the local database of the Institute for Pharmaceutical Biology and Biotechnology, Heinrich Heine University, Düsseldorf, Germany. The analyzed samples were the crude extract of *P. americana* (PA) and its fractions (PAH, PAE, PAM, and PAW).

### 2.11. Statistical Analysis

Data analysis was done using IBM SPSS, version 21.0 package. The results were expressed as mean ± standard error of mean (SEM). One-way analysis of variance (ANOVA) with Dunnett's test for multiple comparisons was used to compare means across the groups. Mean values with *p* < 0.05 were considered statistically significant compared to the control group.

## 3. Results

### 3.1. Results of Acute Toxicity Test

The results of the acute toxicity study (Table 1) show that there was no mortality at the various doses tested for *P. americana*. This indicates that the LD_50_ is greater than 5000 mg/kg. There were no visible signs of overt toxicity such as tremors, loss of appetite, lacrimation, diarrhea, or salivation within 14 days of physical examination. However, mortality occurred at 5000 mg/kg of *D. edulis* extract. Hence, the LD_50_ for *D. edulis* was calculated to be 4243 mg/kg. These results formed the choice of the doses used for the extracts and fractions.

### 3.2. Rane's Curative Test Model

The results of the antimalarial test based on Rane's curative model (Table 2) show that both extracts (PA and DE) produced dose-dependent inhibition (*p* < 0.05) of parasitemia. The inhibition produced by 400 mg/kg of *D. edulis* was 57.10 ± 1.90% while the same dose of *P. americana* produced a comparable but slightly lower inhibition of 52.16 ± 2.77%. Their effect was also comparable, a little lower than that of the standard drug, ACT (69.04 ± 3.02% inhibition).

### 3.3. Hematological Parameters in Rane's Curative Model

The results of Hb and PCV are shown in Table 3. Results show that there was a reduction of the hematological parameters as a result of the infection with the parasite from day 0 to day 3. The plant extracts improved (*p* < 0.05) these parameters after the 5-day treatment period. The results of Hb show that the two extracts produced a dose-dependent significant (*p* < 0.05) increase in the Hb from day 3 (the day of infection) to day 8 (a day after treatment) in the curative test model. The increment for *P. americana* ranges from 5.84 ± 0.08% to 33.94 ± 0.12% while that of *D. edulis* ranges from 22.54 ± 0.14% to 39.23 ± 0.25%. The increment values are however less than those of ACT (43.11 ± 0.17%). Similarly, the results of the PCV shows that both extracts also produced significant increment in the PCV of the parasitized mice though that of *P. americana* was in a non-dose-dependent manner (maximum increment was 26.06 ± 0.07%). *D. edulis* produced dose-dependent increment ranging from 4.69 ± 0.80% to 13. 04 ± 0.33%.

### 3.4. Suppressive Antimalarial Test Model

The results of the suppressive antimalarial test model, mean survival time, body weight, and PCV are shown in Table 4. The *P. americana* extract at 400 mg/kg produced significant (*p* < 0.05) chemosuppression (55.00 ± 0.06%) in parasitemia. Similarly, the hexane (PAH), ethyl acetate (PAE), and water (PAW) fractions produced significant (*p* < 0.05) suppression by 56.03 ± 0.07%, 40.00 ± 0.05%, and 64.01 ± 0.08%, respectively. The rank order of chemosuppression of the fractions was water (64.01 ± 0.08%) > hexane (56.03 ± 0.07%) > ethyl acetate (40.00 ± 0.05%) > methanol (22.02 ± 0.09%). Likewise, the extract and fractions of *D. edulis* produced a significant (*p* < 0.05) chemosuppression of parasitemia. The extract of *D. edulis* at the dose of 400 mg/kg produced (suppression of 62.03 ± 0.01%) slightly better effect than *P. americana* extract (55.00 ± 0.06%) at the same dose. Also, the fractions of *D. edulis* showed chemosuppression ranging from 53.51 ± 0.09% (for the hexane fraction) to 71.99 ± 0.06% (for methanol fraction). The effect of the latter is slightly better than that of ACT (70.00 ± 0.06%).

#### 3.4.1. Survival Time in the Suppressive Antimalarial Test Model

While the untreated group (control group) caused a mean survival time of only 4.8 ± 0.66 days, the extract and fractions of *P. americana* prolonged the survival times in the range of 5.80 ± 1.69 to 17.6 ± 3.67 days with only the water fraction producing a significant (*p* < 0.001) effect (Table 4). In addition, *D. edulis* extract and fractions prolonged the survival time of the test animals in the range of 8.0 ± 4.29 to 11.60 ± 5.07 days with the methanol fraction producing the longest surviving time.

### 3.5. Body Weight and PCV in the Suppressive Antimalarial Test Model

Though there was a general decline in body weight in all the groups that received *P. americana* extract and fractions, there was a reduction of 18.22 ± 0.07% in the body weight in the control group while, for the hexane fraction, there was a moderate reduction of 6.25 ± 0.04%; ACT has an increment of 14.44 ± 0.02% (Table 4). None of the fractions and the extract produced a significant (*p* > 0.05) change in the PCV of the animals in the suppressive model within the 5-day observation period (day 0 to day 4) though the ethyl acetate and methanol fractions produced moderate increment in the parameter. For the *D. edulis*, there was a significant (*p* < 0.05) increase in body weight due to the effect of the extract and fractions in the range of 11.05 ± 0.03% to 28.55 ± 0.07%. Similar to *P. americana*, the extract and fractions of *D. edulis* did not produce significant (*p* > 0.05) change in the PCV of the mice in the suppressive model.

### 3.6. Results of Phytochemical Analysis

Results of phytochemical analysis (Table 5) revealed the presence of all the tested phytoconstituents like alkaloids, saponins, tannins, flavonoids, steroids, and glycosides in both crude leaf extracts of *P. americana* and *D. edulis*. All the plant samples contain saponins. As shown in Table 5, alkaloids are also present in the moderately polar fractions of both plants.

### 3.7. Results of HPLC Analysis

**Figure 1:**
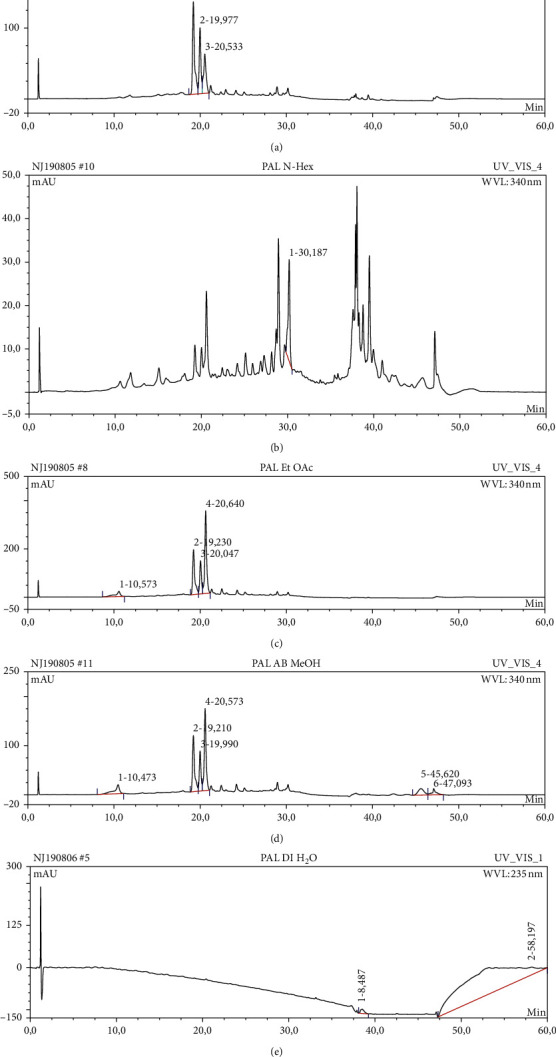
HPLC chromatograms of *P. americana* extract and fractions ((a) PA, (b) PAH, (c) PAE, (d) PAM, (e) PAW).

The results of HPLC analysis (Figure 1 and Table 6) suggested the presence of flavonoids, alkaloids, fatty acids, steroids, or terpenes as the major phytoconstituents in the extract and fractions of *P. americana* leaf. The major constituents of the hexane fraction (PAH) are steroids/terpenes and fatty acids while the moderately polar fraction (ethyl acetate) contains more flavonoids. The polar fractions (methanol and water fractions) contain mainly flavonoids and alkaloids. Quercetin and its glycosides are the most abundant flavonoids identified in the plant.

## 4. Discussion

Toxicological investigation of the extracts was done to ascertain their safety for use in the animals with possible extension to humans. LD_50_ is used as an indication of acute toxicity [20, 21]. High values of the LD_50_ (>5000 mg/kg for *P. americana* and 4243 mg/kg for *D. edulis*) obtained from the present work indicate the safety of the leaf extracts of the plants and hence formed the bases for the doses chosen for the antimalarial studies. Further support for the safety of these plants is the fact that their fruits are edible and are used for both nutritional and therapeutic purposes [26]. However, some toxic compounds have been identified in the seed of *D. edulis* seeds; otherwise, the plant is considered to be safe [27]. In addition, in line with the present work, Kamagate et al. [28] reported the LD_50_ of *P. americana* leaf as greater than 5000 mg/kg.

The present study employed *in vivo* antiplasmodial model in rodents since the model takes into account the possible involvement of the immune system and possible prodrug effect in the activity of the plants against the parasites [29]. Moreover, several antimalarial drugs such as artemisinin derivatives and chloroquine have been identified using in vivo model [30]. Both curative and suppressive models were employed in order to establish the curative capability on established infection as well as the schizonticidal activity of the plants. In both models, the mean parasitemia level was lower than 90% which indicates that both plants were active as antimalarial agents [31]. Antimalarial agents are expected to decreased parasitemia and its symptoms. This can be achieved through various means like reducing parasite nutrient intake and interfering with the pathways such as the heme pathway [32] or they could negatively affect the growth and reproduction of parasites [33]. The extracts from both *P. americana* and *D. edulis* reduced parasitemia in both the curative and suppressive models and made some of the mice survive for a longer time. Comparatively, in both antimalarial models, *D. edulis* extract produced a slightly better effect (57.10 ± 1.98% inhibition in the curative model and 62.03 ± 0.01% chemosuppression) than *P. americana* extract (52.16 ± 2.77% inhibition in the curative model and 55.00 ± 0.06% chemosuppression) but their effects are somewhat lower than that of the ACT (69.04 ± 3.02% inhibition in the curative model and 70.00 ± 0.06% chemosuppression).

Likewise, the effects of the fractions further indicate the antimalarial activity of both plants as all the tested fractions (besides the methanol fraction of *P. americana*) from both plants produced (*p* < 0.05) chemosuppression. The antimalarial activity of *D. edulis* appears to concentrate more on the polar fraction (methanol fraction produced 71.99 ± 0.06% chemosuppression) than the nonpolar fractions. Similarly, the polar (water) fraction of *P. americana* produced a better effect (64.01 ± 0.08% chemosuppression) than others. Thus, the methanol fraction of *D. edulis* produced the largest parasitemia suppression (71.99 ± 0.06%) among all the tested extracts and fractions of both plants. The results of the present study validate the use of both plants, either singly or in combination in ethnomedicinal practice for the treatment of malaria [12–14, 17].

The decrease in PCV and Hb of the malarial-infected mice is in conformity with previous reports in malarial subjects [34]. In the present study, the leaf extracts of both plants in Rane's curative model caused a significant increase in Hb and PCV as a result of increased production of RBC. Body weight increase, particularly by *D. edulis* extract, suggests that the extracts and fractions have an ameliorating effect on the malaria infection. Besides, the prolongation of the surviving times of the infected mice by the extract and fractions of both plants is a further proof of the strong antimalarial potential of the plants.

The observed antimalarial activity of both plants could be attributed to their phytochemical constituents including alkaloids, flavonoids, saponins, tannins, steroids, or glycosides either acting singly or synergistically as observed from the phytochemical and HPLC analysis. Some authors have previously isolated and identified some compounds having antimalarial activity in some parts of the plants. In a previous report, some compounds were isolated from *D. edulis* stem bark and shown to have a promising effect against *P. falciparum* with the most active compound being methyl 3, 4, 5-trihydroxybenzoate [17]. Compounds which were reportedly present in *P. americana* include peptone, b-galactoside, alkaloids, glycosylated abscisic acid, saponins, polyphenols, and fatty alcohols [29, 35, 36].

Our data indicate the presence of the antimalarial constituent of the leaf extract of *P. americana* in the water, hexane, and ethyl acetate fractions since these fractions produced significant (*p* < 0.05) suppression of parasitemia. The phytoconstituents of these fractions were varied and include saponins and tannins (for water fraction); saponins and steroids (for hexane fraction); and alkaloids, saponins, and tannins (for ethyl acetate fraction). HPLC analysis revealed the presence of main flavonoids in *P. americana* particularly quercetin and its glycosides. These results, therefore, suggest that the antimalarial constituents of *P. americana* are varied in nature. Likewise, methanol fraction, which was the most active fraction of *D. edulis,* contained alkaloids, saponins, tannins, flavonoids, and glycosides. One or more of these compounds could be responsible for the antimalarial activity of the plant [32, 37]. Thus, a higher concentration of these phytoconstituents in the active fractions could also be responsible for their high antimalarial property.

Various studies have supported the antimalarial activity of these plant constituents. Alkaloids, flavonoids, and sesquiterpenes have been reported to possess a broad spectrum of bioactivities including antimalarial activity [38, 39]. Many alkaloids, in particular, possess antimalarial activity and this is exemplified by quinine which is one of the most important and oldest antimalarial drugs [40, 41]. Other phytoconstituents including saponins, steroids, flavonoids [41], and terpenoids [42] have been reported to exhibit potent antimalarial activity. These previous reports are further support for the antimalarial activity of *P. americana* and *D. edulis* since these phytochemicals were identified in these studied plants.

Several mechanisms of action have been postulated for various natural antiplasmodial agents. Quercetin, a flavonoid, was shown to exhibit its antiparasitic effect through the destruction of mitochondrial function and the inhibition of different important enzymes and molecules, including heat-shock protein (HSP), acetylcholinesterase, DNA topoisomerase, and kinase [43]. Also, prenylated chalcones isolated from *Humulus lupulus* were reported to interfere with ham degradation in *P. falciparum*, suggesting a possible mechanism of action [44]. Flavonolignans and catechins also inhibit *β*-haematin formation in the parasite similar to chloroquine and H_2_O_2_-mediated heme degradation [45]. Some bromopyrrole alkaloids inhibited *Plasmodium* type II fatty acid synthase (FAS II) enzyme, suggesting that this might be part of the mechanism of action [46]. Also, some bromophycolides inhibited haemozoin formation by targeting heme crystallization [47]. Tajuddeen and Van Heerden [48] reviewed several other antiplasmodial mechanisms of action for natural compounds, including production of reactive oxygen species (ROS) and lipid oxidation products, inhibition of food vacuole falcipains, and *Plasmodium falciparum* glyoxalase I (PfGLOI), among others. These authors [48] also identified several natural compounds and medicinal plants that have been reported to exhibit a transmission-blocking effect by gametocidal activity. Though the actual mode of action of the antiplasmodial action of the plants was not studied in this present report, it is possible that the plants could exhibit their activity by some of the reported mechanisms of action.

The results of the present study provide justification for the anecdotal use of the two plants in the traditional medicines for the treatment of malaria. Further works are also underway to isolate and characterize the antimalarial constituents of the plants.

## 5. Conclusions

Herein, the activity of the leaf extracts and fractions of *P. americana* and *D. edulis* against malaria parasite in both curative and suppressive mouse models has been demonstrated. In addition, the extracts and fractions of the plants prolonged the survival time of the infected mice while also improving their hematological parameters. The most active antimalarial fractions of the two plants are the more polar fractions. The study provides further scientific justification for the use of the two plants in Nigerian and other ethnomedicines for the treatment of malaria. Additionally, based on our results, the leaf extracts may be a source for potential novel compounds against malaria parasite. Further studies geared towards the identification of the active compounds responsible for the antimalarial activity observed with the plants are envisaged.

## Figures and Tables

**Table 1:** Results of the acute toxicity studies of the extracts of *P. americana* and *D. edulis*.

Experiment	Number of animals	Extract		
		Dose (mg/kg)	PA	DE
			Mortality rate	
Phase I	3	10	0/3	0/3
	3	100	0/3	0/3
	3	1000	0/3	0/3

Phase II	1	1600	0/1	0/1
	1	2900	0/1	0/1
	1	3600	0/1	0/1
	1	5000	0/1	1/1

**LD**_**50**_ **(mg/kg)**			>5000	4243

**Table 2:** Effect of the extracts on parasitemia of *P. berghei*-infected mice in Rane's curative antimalarial model.

Group	Treatment	% parasitemia		% inhibition of parasitemia
		Day 3	Day 8	
1	100 mg/kg PA	70.80 ± 3.71	46.80 ± 3.44^*∗∗∗*^	33.90 ± 2.13^*∗∗∗*^
2	200 mg/kg PA	69.80 ± 3.54	41.80 ± 4.85^*∗∗∗*^	40.11 ± 5.10^*∗∗∗*^
3	400 mg/kg PA	69.40 ± 1.44	33.20 ± 4.91^*∗∗∗*^	52.16 ± 2.77^*∗∗∗*^
4	100 mg/kg DE	73.40 ± 1.50	66.60 ± 2.46^*∗∗∗*^	9.26 ± 0.08^*∗*^
5	200 mg/kg DE	67.00 ± 3.51	34.20 ± 3.18^*∗∗∗*^	48.96 ± 2.11^*∗∗∗*^
6	400 mg/kg DE	60.60 ± 2.94	26.00 ± 2.05^*∗∗∗*^	57.10 ± 1.98^*∗∗∗*^
7	ACT	78.80 ± 3.58	24.40 ± 5.02^*∗∗∗*^	69.04 ± 3.02^*∗∗∗*^
8	Control	71.20 ± 2.47	79.60 ± 5.4	−11.80 ± 1.07

Data are mean ± standard error of mean (SEM) (*n* = 5); ^*∗*^*p* < 0.05, ^*∗∗*^*p* < 0.01, ^*∗∗∗*^*p* < 0.001 as compared with control group; % inhibition of parasitemia = (% parasitemia on day 3 − % parasitemia on day 8/% parasitemia on day 3) × 100; DE = *D. edulis* leaf extract; PA = *P. americana* leaf extract.

**Table 3:** Effect of the extracts on the Hb and PCV of *P. berghei*-infected mice in Rane's curative antimalarial model.

Group	Treatment	Hb (mg/dL)				PCV (%)			
		Day 0	Day 3	Day 8	% change in Hb^#^	Day 0	Day 3	Day 8	% change in PCV^#^
1	100 mg/kg PA	11.00 ± 0.25	9.24 ± 0.11^*∗*^	9.78 ± 0.15^*∗∗∗*^	5.84 ± 0.08	40.80 ± 0.58	30.80 ± 1.85	35.80 ± 0.92^*∗∗∗*^	16.23 ± 0.06
2	200 mg/kg PA	11.26 ± 0.23	8.42 ± 0.61^*∗*^	10.28 ± 0.21^*∗∗∗*^	22.09 ± 0.23	41.20 ± 0.80	28.40 ± 1.21	35.80 ± 0.66^*∗∗∗*^	26.06 ± 0.07
3	400 mg/kg PA	11.40 ± 0.27	7.72 ± 0.30^*∗*^	10.34 ± 0.18^*∗∗∗*^	33.94 ± 0.12	41.80 ± 1.36	31.60 ± 0.93	36.80 ± 1.16^*∗∗∗*^	16.46 ± 0.40
4	100 mg/kg DE	11.08 ± 0.13^*∗∗*^	7.72 ± 0.29^*∗*^	9.46 ± 0.24^*∗*^	22.54 ± 0.14	42.25 ± 1.10	32.00 ± 1.08	33.50 ± 0.95^*∗∗∗*^	4.69 ± 0.80
5	200 mg/kg DE	11.20 ± 0.12^*∗*^	8.46 ± 0.63^*∗*^	10.50 ± 0.12^*∗∗∗*^	24.11 ± 0.23	42.20 ± 0.80	32.80 ± 1.66	35.80 ± 0.73^*∗∗∗*^	9.15 ± 0.90
6	400 mg/kg DE	11.00 ± 0.09^*∗*^	7.80 ± 0.36^*∗*^	10.86 ± 0.19^*∗∗∗*^	39.23 ± 0.25	40.40 ± 0.68	32.20 ± 1.56	36.40 ± 0.68^*∗∗∗*^	13.04 ± 0.33
7	Standard drug (ACT)	11.28 ± 0.71	7.84 ± 0.29	11.22 ± 0.13^*∗∗∗*^	43.11 ± 0.17	41.40 ± 0.74	32.60 ± 1.60	39.80 ± 0.66^*∗∗∗*^	22.09 ± 0.87
8	Control (infected but untreated)	10.98 ± 0.13	7.06 ± 0.28	6.90 ± 0.31	−2.27 ± 0.05	41.80 ± 0.58	28.60 ± 1.32	24.00 ± 1.44	−16.08 ± 0.05

Data are mean ± standard error of mean (SEM) (*n* = 5); ^*∗*^*p* < 0.05, ^*∗∗*^*p* < 0.01, ^*∗∗∗*^*p* < 0.001 as compared with control group; ^#^% change in Hb or PCV is that of day 3 and day 8.

**Table 4:** Parasitemia, survival time, body weight, and PCV of infected mice treated with the extracts and fractions in the four-day suppressive test.

Group	Treatment dose (mg/kg)	Parasitemia level		Mean survival time (days)	Body weight (g)			PCV (%)		
		% parasitemia	% suppression		Day 0	Day 4	% change	Day 0	Day 4	% change
1	400 mg/kg PA	7.50 ± 0.65^*∗∗∗*^	55.00 ± 0.06	6.60 ± 1.08	12.44 ± 1.44	10.64 ± 0.52	−14.47 ± 0.06	41.00 ± 1.00	37.25 ± 0.75	−9.15 ± 0.03
2	200 mg/kg PAH	7.33 ± 0.88^*∗∗∗*^	56.03 ± 0.07	6.00 ± 1.52	11.84 ± 0.91	11.10 ± 0.80	−6.25 ± 0.04	39.00 ± 0.71	37.01 ± 0.58	−5.10 ± 0.02
3	200 mg/kg PAE	10.00 ± 0.58^*∗*^	40.00 ± 0.05	5.80 ± 1.69	15.48 ± 2.89	12.75 ± 2.08	−17.64 ± 0.01	38.20 ± 0.66	39.67 ± 1.45	3.85 ± 0.03
4	200 mg/kg PAM	13.00 ± 1.41	22.02 ± 0.09	10.6 ± 2.73	14.10 ± 2.27	12.36 ± 1.62	−12.34 ± 0.03	38.80 ± 0.86	39.25 ± 0.63	1.16 ± 0.04
5	200 mg/kg PAW	6.01 ± 0.91^*∗∗∗*^	64.01 ± 0.08	17.6 ± 3.67^*∗∗∗*^	13.82 ± 1.32	12.16 ± 1.10	−12.01 ± 0.01	39.40 ± 0.60	37.75 ± 0.63	−4.19 ± 0.02
6	400 mg/kg DE	6.33 ± 0.33^*∗∗∗*^	62.03 ± 0.01	10.20 ± 2.97	19.76 ± 1.47	20.80 ± 0.48^*∗∗*^	5.26 ± 0.03	40.00 ± 0.32	36.33 ± 2.19	−9.18 ± 0.02
7	200 mg/kg DEH	7.75 ± 1.25^*∗∗∗*^	53.51 ± 0.09	10.40 ± 4.15	16.88 ± 0.69	21.70 ± 2.71^*∗∗*^	28.55 ± 0.07	39.40 ± 1.08	36.50 ± 1.70	−7.36 ± 0.12
8	200 mg/kg DEE	7.00 ± 0.58^*∗∗∗*^	58.01 ± 0.06	8.0 ± 4.29	19.36 ± 0.60	21.50 ± 0.15^*∗∗*^	11.05 ± 0.03	38.20 ± 0.66	34.00 ± 0.58	−10.99 ± 0.05
9	200 mg/kg DEM	4.67 ± 0.88^*∗∗∗*^	71.99 ± 0.06	11.60 ± 5.07	17.32 ± 1.02	19.78 ± 0.94^*∗∗*^	12.44 ± 0.02	40.60 ± 0.51	34.00 ± 0.58	−16.26 ± 0.04
10	ACT	5.01 ± 0.58^*∗∗∗*^	70.00 ± 0.06	16 ± 1.14^*∗∗∗*^	17.52 ± 0.47	20.05 ± 0.52	14.44 ± 0.02	39.40 ± 0.51	39.67 ± 0.33	0.69 ± 0.02
11	Control	16.67 ± 1.45	—	4.8 ± 0.66	18.22 ± 1.69	14.90 ± 1.22	−18.22 ± 0.07	40.00 ± 0.71	36.67 ± 1.76	−8.33 ± 0.08

PA = *P. americana* leaf crude extract, PAH = *P. americana* hexane fraction; PAE = *P. americana* ethyl acetate fraction*;* PAM : *P. americana* methanol fraction; PAW *=* *P. americana* water fraction; DE = *D. edulis* leaf extract; DEH = *D. edulis* hexane fraction; DEE = *D. edulis* ethyl acetate fraction; DEM = *D. edulis* methanol fraction. DEW (*D. edulis* water fraction) was not tested due to its limited quantity.

**Table 5:** Results of phytochemical analysis of the leaf extract and fractions of *P. americana* and *D. edulis*.

Phytochemicals	PA	PAH	PAE	PAM	PAW	DE	DEH	DEE	DEM
Alkaloids	**+**	−	**+**	**+**	−	+	+	+	+
Saponins	**+**	**+**	**+**	**+**	**+**	+	+	+	+
Tannins	**+**	−	**+**	**+**	**+**	+	−	+	+
Flavonoids	**+**	−	−	**+**	−	+	−	−	+
Steroids	**+**	**+**	−	−	−	+	+	+	−
Glycosides	**+**	−	−	**+**	−	+	−	+	+

PA = *P. americana* leaf crude extract, PAH = *P. americana* hexane fraction; PAE = *P. americana* ethyl acetate fraction*;* PAM : *P. americana* methanol fraction*;* PAW *=* *P. americana* water fraction; DE = *D. edulis* leaf crude extract; DEH = *D. edulis* hexane fraction; DEE = *D. edulis* ethyl acetate fraction; DEM = *D. edulis* methanol fraction; + = present; − = absent.

**Table 6:** Results of HPLC analysis of *P. americana* extract and its fractions.

Sample	Ret. time (min)	% peak area	UV maxima (rel. int.)	Suggested name of compound	class of phytochemical
PA	12.65	17.39	239.6 (50), 321.1 (5), 357.7 (5)	Cytosporin	Flavonoids
	19.203	36.00	202.9 (50), 257.0 (30), 356.5 (45)	Hyperoside (quercetin-3-galactoside)	Flavonoids
	19.977	19.08	203.2 (50), 256.7 (30), 356.5 (45)	Quercetin-3-O-galactoside	Flavonoids
	20.533	21.47	201.8 (50), 255.9 (30), 349.7 (45)	Quercetin-3-O-rhamnoside	Flavonoids
	47.08	6.06	201.8 (50), 280.4 (10), 371.7 (10)	Cyclopenol	Alkaloids

PAH	12.62	9.80	237.9 (50), 350.4 (10)	Cyteo-*α*-pyrone	Pyrones
	20.58	4.28	204.0 (50), 255.0 (30), 350.5 (20)	Quercetin-3-O-rhamnoside	Flavonoids
	31.88	10.99	233.9 (50), 277.8 (5)	Fatty acid	Fatty acid
	32.03	16.42	234.2 (50), 274.3 (5)	Fatty acid	Fatty acid
	33.05	12.14	232.9 (50), 278.5 (5)	Cerebroside	Steroids/terpenes
	40.89	4.38	251.8 (50), 405.9 (10)	*β*-Sitosterol-3-O-*α*-pyranoside	Steroids
	47.07	19.56	202.4 (50), 280.3 (10), 371.6 (10)	Pretrichodermamide	Alkaloids

PAE	19.23	6.73	203.7 (50), 257.0 (30), 356.6 (30)	Hyperin	Flavonoids
	20.05	4.12	203.6 (50), 256.8 (30), 356.6 (30)	Quercetin-3-O-galactoside	Flavonoids
	20.64	12.78	204.0 (50), 257.1 (30), 350.1 (30)	Quercetin	Flavonoids

PAM	19.21	21.40	203.2 (50), 257.1 (30), 356.7 (45)	Hyperoside (quercetin-3-galactoside)	Flavonoids
	19.99	11.90	203.2 (50), 256.9 (30), 356.7 (45)	Hyperoside (quercetin-3-galactoside)	Flavonoids
	20.57	27.75	204.0 (50), 257.1 (30), 350.1 (30)	Quercetin	Flavonoids

PAW	38.49	1.05	201.2 (50), 226.1 (30), 276.2 (30)	Naamine	Alkaloids

PA = *P. americana* leaf crude extract; PAH = *P. americana* hexane fraction; PAE = *P. americana* ethyl acetate fraction*;* PAM : *P. americana* methanol fraction*;* PAW *=* *P. americana* water fraction.

## Data Availability

The data used to support the findings of this study are available from the corresponding author upon request.
